# A Discussion of a Case of Paradoxical Ipsilateral Hemiparesis in a Patient Diagnosed with Pterional Meningioma

**DOI:** 10.3390/jcm14082689

**Published:** 2025-04-15

**Authors:** Ligia Gabriela Tataranu

**Affiliations:** 1 Department of Neurosurgery, Carol Davila University of Medicine and Pharmacy, Bucharest 020021, Romania; ligia.tataranu@umfcd.ro; 2 Department of Neurosurgery, Bagdasar-Arseni Emergency Clinical Hospital, Bucharest 041915, Romania

**Keywords:** ipsilateral hemiparesis, Kernohan-Woltman notch phenomenon, Ectors’ syndrome, meningioma, neurosurgery

## Abstract

**Background:** Although various theories have been developed to explain ipsilateral hemiparesis, the causes behind this clinical phenomenon are still poorly understood. The main pathophysiological hypotheses include the anatomical variations in decussation of the corticospinal tract, the theory of diaschisis, the Kernohan-Woltman notch phenomenon, and Ectors’ syndrome. The current article aims to report the case of a 43-year-old woman diagnosed with ipsilateral hemiparesis following a right pterional meningioma, later treated by surgery. The different theories behind this paradoxical clinical phenomenon are discussed to elucidate the most likely mechanism behind it. **Methods:** A 43-year-old right-handed woman with a history of splenomegaly and iron deficiency anemia was admitted to our hospital for refractory headache, right-sided hemiparesis, and generalized tonic-clonic seizures. Clinical examination revealed a right upper motor neuron syndrome, with a grade 4 MRCS muscle strength for the upper and lower limbs. The contrast-enhanced brain MRI revealed an extra-axial right pterional mass lesion with a broad dural base, well-defined margins, and intense post-contrast enhancement, suggestive of meningioma. The patient was surgically treated for the brain lesion. **Results:** After a Simpson grade I resection with complete removal of the tumor and affected dura, the patient had a favorable local and neurological evolution, and after three weeks, total remission of the symptoms was achieved. **Conclusions:** To assess the mechanism behind ipsilateral hemiparesis, thorough clinical examination and further research in neuroimaging assessment and functional studies are essential.

## 1. Introduction

Ipsilateral hemiparesis (IH) is a paradoxical clinical entity characterized by an upper motor neuron disorder affecting one side of the body contralateral to those expected, given the topography of the triggering intracranial lesion [1]; therefore, weakness occurs on the same side as brain lesions. This clinical manifestation runs contrary to the classical known model of contralateral motor control based on corticospinal tract (CST) decussation, not only puzzling the physicians but also adding to the intrigue surrounding the complexity of neural pathways. Furthermore, it opens a window into the resiliency and plasticity of the human nervous system by anatomical substratum, clinical presentation, and therapeutic developments [2]. The primary objective of the current manuscript is to chronicle different etiological theories for IH while creating an interesting discussion around the presented case.

## 2. The Case: An Intriguing Case Unraveling Clinical and Paraclinical Complexities

A 43-year-old right-handed woman with a history of splenomegaly and iron deficiency anemia was admitted to our hospital for refractory headache, right-sided hemiparesis, and generalized tonic-clonic seizures. The onset of these symptoms was approximately 4 months before hospital admission and worsened over time. Clinical examination assessed the muscle strength according to the modified Medical Research Council Scale (MRCS). A grade 4 muscle strength (active movement against moderate resistance) was determined for the right upper and lower limbs. Right deep tendon reflexes were graded as 3+ (brisk response), and a right positive Babinski sign was noted. Thus, a right upper motor neuron syndrome was concluded (Table 1).

The inflammatory markers and coagulation studies were within our laboratory’s normal range (Table 2). Furthermore, the patient had no history of metabolic abnormalities, and no current modifications were detected.

**Figure 1:**
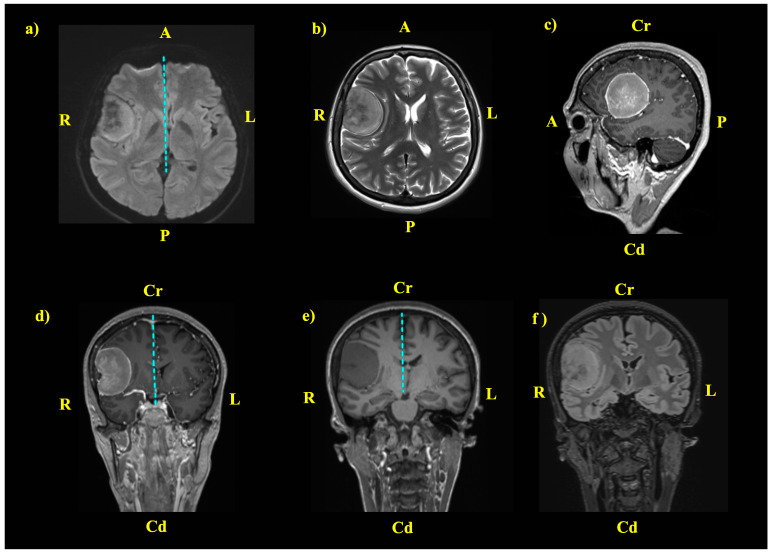
Preoperative contrast-enhanced brain MRI revealing a voluminous pterional space-occupying lesion. (**a**) TRACE transverse relaxation time axial T2-weighted sequence; (**b**) BLADE axial T2-weighted sequence; (**c**) sagittal T1 contrast-enhanced sequence; (**d**) coronal T1 contrast-enhanced sequence; (**e**) coronal T1-weighted sequence; (**f**) T2 dark fluid sequence, coronal view; A—anterior; P—posterior; Cr—cranial; Cd—caudal; R—right; L—left. The blue dotted line highlights the midline shift, which is more evident in selected projections.

A contrast-enhanced brain MRI was performed, which revealed an extra-axial right pterional mass lesion with a broad dural base, well-defined margins, and a cerebrospinal fluid (CSF) cleft sign, spontaneously isodense to grey matter on T1-weighted images and slightly hyperintense on T2-weighted images and Fluid-Attenuated Inversion Recovery (FLAIR) images. The mass showed intense post-contrast enhancement with a heterogeneous pattern. The tumor had an anteroposterior diameter of 52 mm, a transversal diameter of 43 mm, and a craniocaudal diameter of 53 mm, and caused a 5 mm midline shift due to the mass effect (Figure 1). The MRI characteristics were suggestive of meningioma. As a standard department policy, informed consent was obtained for all procedures, including the publication of the case under the relevant anonymization rules.

**Figure 2:**
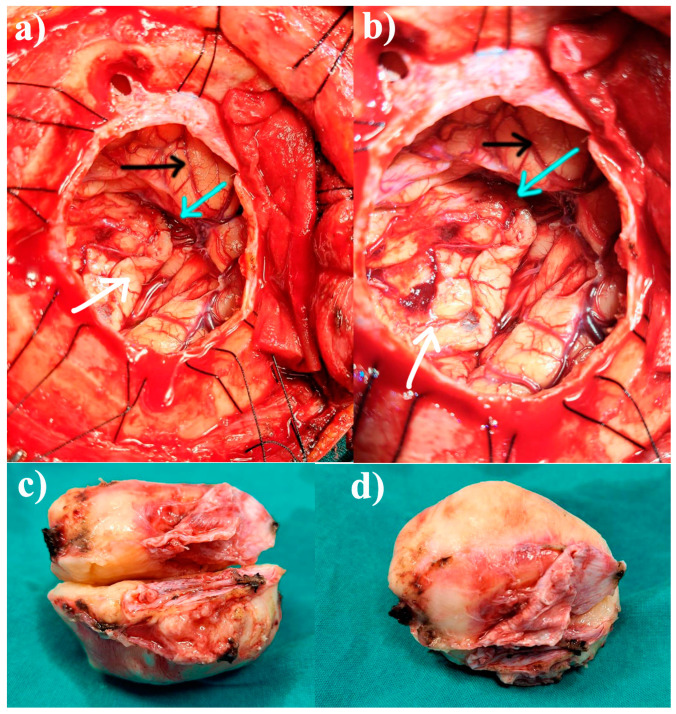
(**a**) Intraoperative aspect after gross total resection centered on the Sylvian fissure. (**b**) Intraoperative aspect after gross total resection centered on the temporal lobe; the blue arrow points to the Sylvian fissure, the black arrow points to the frontal lobe, and the white arrow points to the temporal lobe. (**c**,**d**) The tumor after surgical excision; macroscopic aspect highlights the typical aspect of the meningioma.

After the preoperative evaluation, the neurosurgical intervention was decided, and the patient gave her written consent. A gross total resection was performed using a right pterional approach under general anesthesia. During the surgery, a tumoral infiltration of the dura mater was observed without bone invasion. After the dural opening, a round voluminous tumoral mass was discovered, well-circumscribed, with a red-greyish color, firm consistency, and intense vascularization, suggestive of meningioma. A Simpson grade I resection was performed, with complete removal of the tumor and affected dura (Figure 2).

**Figure 3:**
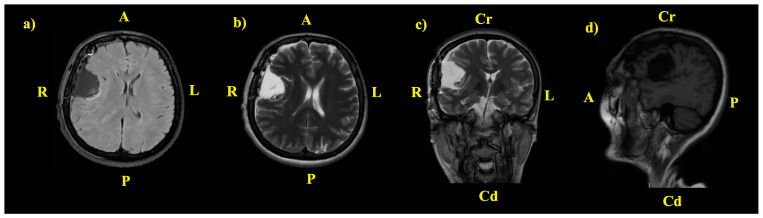
Postoperative brain MRI; A—anterior; P—posterior; Cr—cranial; Cd—caudal; R—right; L—left. (**a**) Axial T2-weighted fluid-attenuated inversion recovery (FLAIR) sequence. (**b**) Axial T2-weighted PROPELLER. (**c**) Coronal T2-weighted fast spin-echo (FSE) sequence. (**d**) Sagittal T1 FLAIR sequence.

After surgery, the patient had a favorable local and neurological evolution. A brain MRI was performed early postoperative (at 48 h), and the results showed the right pterional craniotomy, with a postsurgical cavity after the tumor removal, measuring 38 mm/28.5 mm/39 mm (anteroposterior/transversal/craniocaudal) and adjacent gliotic changes. The midline shift was still persistent (Figure 3).

**Figure 4:**
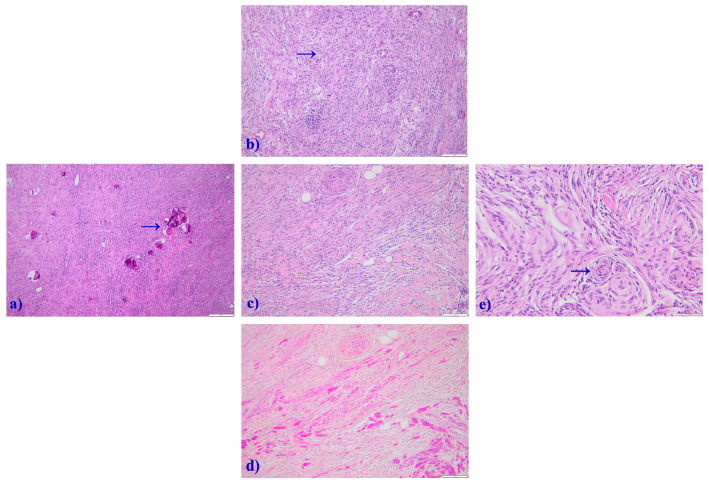
Photomicrographs depicting histopathologic features specific to intracranial transitional meningioma WHO grade I; (**a**) Hematoxylin-Eosin staining showing psammoma bodies (arrow) under 10× magnification. (**b**) Hematoxylin-Eosin staining showing cellular whorls (arrow) under 20× magnification. (**c**) Hematoxylin-Eosin staining showing associated extensive collagen deposition under 20× magnification. (**d**) Van Gieson staining showing associated extensive collagen deposition under 20× magnification. (**e**) Hematoxylin-Eosin staining showing cellular whorls (arrow) under 40× magnification.

The histopathological examination concluded a diagnosis of transitional meningioma WHO grade 1 (Figure 4).

Three weeks after the surgery, the presenting symptoms (headache and right-sided hemiparesis) were in total remission. The seizures did not recur, and the evolution was favorable, with no clinical incidences.

## 3. Discussion

Opposing the classic variant of contralateral motor impairment caused by pyramidal tract decussation in the medulla, IH is a paradoxical entity that might involve partial or complete uncrossed CST, as well as mechanical compression or injury in the contralateral neurological structures, such as crus cerebri. Neuroimaging techniques can reveal the intricacies behind this rare phenomenon and, along with clinical history and physical examination, can provide a complete diagnosis. This case report aims to describe the uncommon case of a young woman with an ipsilateral motor deficit caused by a tumoral lesion. Such presented cases are to be considered, given the scarce information regarding this subject, and they can contribute to a better understanding of the underlying mechanisms of this clinical phenomenon. The main pathophysiological hypotheses include disorders of anatomical decussation of the CST, the theory of diaschisis, the Kernohan-Woltman notch phenomenon, and Ectors’ syndrome.

### 3.1. Pathophysiological Hypotheses

#### 3.1.1. Disorders of Anatomical Decussation

The pyramidal tract is the main motor pathway that carries signals for voluntary movement. The system originates mainly in the primary motor cortex and in premotor areas, where upper motor neurons are located [2]. Their axons project from the cortex to the spinal cord (CST) or brainstem (corticonuclear tract) to form synapses onto the lower motor neurons, which carry efferent signals directly to the muscles. According to anatomical terminology, the term “pyramidal tract” refers to only the CST, but in everyday practice, this designation is used for both tracts [2]. The importance of the CST course from the clinical neurology point of view is paramount, given that lesions could affect fibers throughout their pathway [2]. Axons of the upper motor neurons descend and converge in the corona radiata, then pass through the internal capsule and the cerebral peduncle to the pons and medulla. At the lower end of the medulla, the majority (75–90%) of the fibers of the CST decussate and form the lateral CST, while a minority of uncrossed fibers remain ipsilateral and form the anterior CST in the spinal cord. The former controls the voluntary movement of the limbs, while the latter provides the voluntary movement of the trunk [2,3,4].

The crossing over is known as pyramidal decussation. It has been stated that CST projects from the cerebral cortex to the spinal cord and represents the most important factor in developing cortical control and movement. Thus, alterations in these structures are responsible for motor deficits [5]. Generally, lesions above the pyramidal decussation produce contralateral weakness, while those below the pyramidal decussation will produce ipsilateral weakness.

IH due to crossing pattern alterations could not be confirmed in vivo in the past. However, in the modern era, these atypical projections are revealed by tractography [6]. Tractography is a 3D MRI technique that allows us to reconstruct the pathways of the main white matter fiber bundles. Moreover, this method can precisely asses the microstructural organization of tracts and characterize the differences that are related to age [6]. Diffusion tensor imaging (DTI) is used to estimate the brain’s axonal organization. This MRI technique can determine the microstructural and physiological changes in the white matter and is used to assess the course and integrity of tracts in vivo [6,7].

Motor-evoked potentials were also deemed successful in these cases. Interestingly, this technique has also demonstrated its utility in identifying impaired conduction of the corticospinal tract during surgery because of dynamic peduncle compression against the tentorial border—exerted involuntarily by surgical manipulation—in patients displaying a preoperative conflict of space in the tentorial hiatus [8,9]. Otherwise, imaging findings have demonstrated that some patients suffering from ipsilateral hemiparesis after brain ischemia display MRI signs of descending Wallerian degeneration of the uncrossed corticospinal tract, providing further evidence to support that pathophysiological hypothesis in vivo [10,11].

Cases of “paradoxical” IH due to a large number of uncrossed fibers of the CST tract have been reported [12,13]. The involvement of ipsilateral structures such as corona radiata [14], internal capsule, basal ganglia, insula, and thalamus were also reported [15]. However, partial uncrossed fibers were not the only fibers reported as a cause of ipsilateral motor deficits.

In a recent case report by Jidal et al., a rare complete uncrossed CST was diagnosed in a 66-year-old man with a left occipital infarct who presented with ipsilateral motor impairment and hemianopsia. After performing DTI, the fiber tracking revealed complete uncrossed pyramidal tracts on either side at the level of the medulla oblongata [16]. In a paper on CST decussation alteration, Cane et al. mentioned a congenital alteration of the tract in Horizontal Gaze Palsy with Progressive Scoliosis (HGPPS) and Congenital Mirror Movements (CMM), stating that in the first case, there is no fiber crossing at all; in contrast, in the second case, a certain amount of fibers will decussate at the pyramidal level [17,18]. Thus, it has been stated that the pyramidal decussation can be aberrant and absent [19].

Anomalous pyramidal tracts may be a result of various brain disorders. Ten Donkellar et al. used the following classification for malformations of the pyramidal tract occurring at various stages of development: (a) malformations of induction; (b) malformations due to abnormal cell proliferation; (c) malformations due to atypical neuronal migration; (d) malformations due to defective mechanisms of axon guidance; and (e) malformations due to secondarily acquired injury that may lead to destructive lesions [20]. Destructive lesions (mainly represented by ischemic strokes) can damage cortical pyramidal neurons or descending projection pathways, and in this case, the crossed collateral projections will remain the primary source of reinnervation [21,22]. Congenital disorders of the CST can lead to a reduction of the tract or even guidance defects associated with abnormal anatomy, resulting in altered midline crossing at the pyramidal decussation or in the spinal cord, sparing the rest of the tract [3]. The uncrossed pyramidal tract was also associated with conditions like encephalocele, Dandy-Walker syndrome, and Apert’s syndrome [3,23]. The disorders associated with complete aplasia of the CST are represented by anencephaly, congenital aqueduct stenosis, and microcephaly. In contrast, disorders associated with the hypoplastic tract are lissencephaly, Walker-Warburg syndrome, and holoprosencephaly. Tract hypoplasia is also present in X-linked disorders, but in these cases, normal pyramidal decussation has been demonstrated [3]. Abnormal CST guidance at the midline has been associated with disorders like CMM, X-linked Kallmann syndrome, and Klippel-Feil syndrome, while mirror movements have been occasionally linked with Joubert, Gorlin, and Möbius syndromes [3,7].

In 2018, Patra et al. described five possible mechanisms that could explain the rare phenomenon of IH caused by an injury of the CST in the supratentorial compartment [24]. The first proposed mechanism included complete non-decussation of the corticospinal fibers. The second mechanism supported the idea that the predominant involvement of the non-decussated fibers forms a significant proportion of the tract. The third proposed mechanism revealed normal tract anatomy and stated that ipsilateral fibers would be recruited over time after an insult on the contralateral side. The last two proposed mechanisms involve the idea of double decussation of the corticospinal fibers in the brainstem and bilateral supply of the supplementary motor cortex [24].

#### 3.1.2. The Concept of Diaschisis

After a localized central nervous system injury, an instant decline in neuronal synaptic functions was observed. While these secondary alterations occur in focal distant areas of the central nervous system, remote from the initial injury, the entire effect is called diaschisis [25].

Notwithstanding the concept of diaschisis was discussed by von Monakow in 1897 in his *Gehirnpathologie*, the author did not use this exact term until 1902, and in 1905 he published his second edition of *Gehirnpathologie* dedicating an entire chapter titled “Shock and diaschisis” [26]. Given his evolving ideas over the years, von Monakow revisited his theory multiple times until 1914, when he described the phenomenon as we know it today. He insisted on using the term diaschisis in certain ways, pointing out that this effect is limited to remote focal alterations, excluding vital functions that remain intact [26]. In the case of less severe lesions in the central nervous system, the functional alteration would recover after the period of diaschisis wears away [1]. This recovery is a passive process and can be more efficient in young patients than in older ones. Monakow stated that this recovery rate is based on the way neurons are linked and the presence of comorbidities, especially regarding circulatory disorders. Furthermore, the described concept comprises the idea that different nervous structures are more sensitive than others and, thus, are more susceptible to diaschisis [26]. It has been stated that diaschisis regresses in phases correlated to neuroanatomical pathways, starting from the lesional site in the brain [27]. In addition, modern neuroimaging studies concluded that changes in metabolism and blood flow in brain areas were considered to be affected, and these alterations showed reduced activity in regions with diaschisis [27]. Neuromodulation through transcranial magnetic stimulation is regarded as a promising therapeutic approach for focal and non-focal distant neurophysiological alterations that are distant from the initial lesion [28,29,30,31].

It is essential to mention that the signs of diaschisis are more apparent in acute brain injuries and are discreet or even absent in chronic and slow-growing lesions. It has been stated that Monakow created this concept to describe acute lesions and later added other terms to describe different types, such as diaschisis corticospinalis, bulbocerebralis, or corticommisuralis [26]. The last one describes the involvement of the corpus callosum, and a lesion in one cerebral hemisphere can negatively impact the other one, which can explain the phenomenon of IH [26]. Another significant type is represented by the transtentorial or crossed cerebellar diaschisis. This occurs after an acute supratentorial injury that will induce a contralateral cerebellar decrease in blood flow and metabolism [25].

The definition of diaschisis has evolved in the last decades. While the initial term described only temporary functional changes of focal intact regions distant to the brain lesion, the term diaschisis is currently used to describe all distant neurophysiological changes related to the lesion, not only the focal ones. The update was supported by the emerging neuroimaging and intricate electrophysiological techniques available nowadays [28]. Therefore, two main types of diaschisis are relevant: focal and non-focal. The focal type is correlated with the initial definition given by von Monakow. It refers to distant circumscribed neurophysiological changes, while the non-focal type refers to widespread changes in structural and functional connectivity between brain areas distant from the lesion [28]. The concept of connectional diaschisis was introduced and refined to characterize all types of distant changes in coupling due to lost afferents from a lesioned node of a defined neuronal network [28,32]. A decrease in interhemispheric functional connectivity between homotopic cortical areas of the motor network was demonstrated in some cases, but many fundamental questions remain unanswered [28]. The diaschisis could be involved in explaining the occurrence of IH.

Noteworthy investigations of diaschisis are represented by diffusion spectrum imaging (DSI), functional MRI, electroencephalography, and magnetoencephalography. The DSI technique can reveal multidirectional fibers and permit fiber tracking along different directions. These will eventually allow better fiber reconstruction, which was not possible with diffusion tensor imaging (DTI), and will further provide insights into the progression of the disease [33]. In addition, functional MRI is a very useful brain-mapping tool that can assess the brain areas involved in specific sensory processing and cognitive functions. At the same time, electrophysiological measures are used to assess electrical potentials and magnetic fluxes that arise from mass neuronal responses within the brain [34].

#### 3.1.3. Kernohan-Woltman Notch Phenomenon (KWNP)

The Kernohan-Woltman Notch Phenomenon is described as a rare neurological circumstance in which an intracranial lesion induces an extensive mass effect resulting in brainstem displacement and compression of the contralateral cerebral peduncle against the free edge of the tentorium, leading to impairment of the descending corticospinal fibers [35]. The consequence of this neurological situation is a “paradoxical” IH [35]. The most common condition resulting in KWNP is a sudden and massive uncal herniation caused by a supratentorial mass lesion.

James Kernohan and Henry Woltman researched IH by analyzing brains with neoplasms from autopsies. As a consequence, in 1929, they published an article with the conclusions, discussing the theory of mechanical compression of the contralateral crus cerebri and the damage of the opposite pyramidal tract as a mechanism that lies behind the ipsilateral motor deficit [36]. The authors also reported the injury of the contralateral pyramidal tract in the cerebral peduncle caused by infratentorial tumoral lesions [36]. Moreover, the authors noted in a small portion of cases with notching of the contralateral crus cerebri the absence of clinical signs of CST injury, but without elucidating the reason behind it [36]. KWNP is considered a false localizing sign and can be a cause of misdiagnosis in individuals with bilateral CST injuries [37]. As the authors initially observed, both supratentorial and infratentorial lesions can cause KWNP [38].

The neuroimaging features and electrophysiological aspects correlated with KWNP have been studied [39,40]. MRI can better reveal compression and structural damage of the crus cerebri contralateral to the mass lesion and provide information about the anatomic extent of the impaired area. Sometimes, surrounding nervous and vascular structures are also involved. In subacute settings, there is evidence of compression ischemic stroke in the contralateral cerebral peduncle with surrounding edema. The lesion is hypointense in the T1-weighted sequence, hyperintense in the T2-weighted and FLAIR sequences, hypointense in the gradient echo sequence, and with restricted diffusion and brightness in the Diffusion-Weighted Imaging (DWI) sequence [40]. Contrast enhancement on T1-weighted imaging may be seen. In the chronic stage, leukomalacic changes could be displayed, with a hypointense signal in the T1-weighted sequence and a hyperintense signal in the T2-weighted and FLAIR sequences [40]. Diffusion Tensor Imaging (DTI) with fiber tracking methods helps visualize the CST, and a narrowing or disruption of fibers could be observed, even in cases with no abnormal signal changes on conventional MRI [41].

It is worth mentioning that in KWNP, the peduncle may become compressed (by persistent lateral displacement of the diencephalic-mesencephalic axis) or contused (by a rapid and reversible lateral displacement of the mentioned axis) against the contralateral tentorial border [42]. Considering the former mechanism, the peduncle undergoes an elastic deformation (produced by the rigid tentorial border) that is sometimes associated with an underlying parenchymal structural lesion [42]. The exact pathophysiological mechanism producing the peduncular lesion mentioned above remains unknown nowadays, as in most instances, MRI findings are insufficient to distinguish between focal contusion and acute ischemia. Thus, it has been demonstrated that the lesion’s MRI signal and morphology are highly variable [43]. Furthermore, a French group of investigators has recently proposed that the peduncular lesion occasionally associated with the KWNP should be included in a spectrum of lesions produced by arterial cerebral ischemia (caused by occlusion of perforators from the posterior cerebral and/or the superior cerebellar arteries) in a clinical-radiological scenario of severe brain displacement and herniation [43].

The physiological abnormalities associated with KWNP can be highlighted by electrophysiological studies, such as transcranial electrical motor evoked potentials (Tc-MEPs) [39]. Lin et al. demonstrated the potential use of positron emission tomography/computed tomography (PET/CT) to provide microstructural and functional information, although this method is not always practical to use [44]. Obtaining and correlating objective diagnostic information about the anatomic and functional status of the descending corticospinal fibers is essential. The analysis of all these aspects could provide correct diagnosis and management, and may serve as a reference for the probability of functional recovery [39,40,44].

KWNP might have several predisposing factors, and this theory has recently been considered. Thus, the grade of mass effect, the degree of brainstem displacement, anatomical variations of the tentorial incisura, and the width of the tentorial notch are considered contributing factors to the development of Kernohan’s phenomenon [45].

#### 3.1.4. The Syndrome of the Third Frontal Convolution

The Belgian neurosurgeon Léon Ectors provided a better understanding of KWNP pathophysiology in his work “*Les meningiomes de la 3me frontale*” from 1945. In this paper, he identifies a specific symptomatology for extra-axial mass lesions located over the third frontal convolution (particularly meningiomas), with a maximum projection onto the inferior frontal gyrus, sometimes extended to the middle frontal gyrus, temporal lobe, or Sylvian fissure [46]. He brought together the characteristic signs and symptoms under the syndrome of the third frontal convolution (later named Ectors’ syndrome). The clinical features included were the following: early IH, frontal mental syndrome, Broadmann area 8 motor syndrome, symptoms of cortical irritation, compression, or raised intracranial pressure, and radiological syndrome. The last one comprises angiographic ingurgitation of the anterior branch of the middle meningeal artery and ventricle displacement. The idea behind this theory is the same as that of KWNP. There are voluminous mass lesions located mainly over the third frontal convolution with significant mass effect, leading to the displacement of the diencephalic-brainstem complex and compression of the contralateral cerebral peduncle against the free edge of the tentorium. IH is caused by damage to the CST [1]. The Belgian neurosurgeon Léon Ectors provided further insight into the pathophysiology of ipsilateral hemiparesis by distinguishing “early” or “precocious” ipsilateral hemiparesis from “late” ipsilateral hemiparesis. The former characteristically develops in the early stages of the disease, involves the leg precociously (which fits with the somatotopy of the corticospinal tract at the cerebral peduncle), and resumes completely after surgical decompression, suggesting elastic deformation of the contralateral cerebral peduncle (without underlying structural lesion) as the more plausible pathological substrate in that setting [1].

Thus, the phenomenon itself was not new. Kernohan and Woltman have previously described it, but Ectors was able to include the IH in a syndrome associated with the presence of meningiomas over the third frontal convolution [1,46].

### 3.2. Discussions of a Rare Encounter: What Is the Possible Cause of IH in Our Case?

After considering all the hypotheses behind IH (Table 3), the current case can be the focus of an entire discussion.

**Figure 5:**
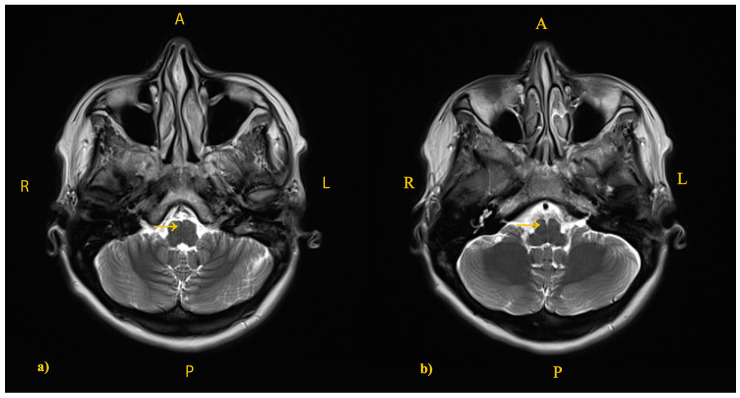
Preoperative brain MRI, T2-weighted axial sequences (**a**,**b**) showing a normal anatomical conformation of the brainstem, suggesting a normal decussation (yellow arrows).

Considering the disorders of anatomical decussation of the CST, it is worth mentioning that MRI techniques are limited in our hospital. Thus, a tractography could not be performed at that given moment. Moreover, given that the patient’s symptomatology was remitted after the neurosurgical excision, she did not agree to perform other investigations in another center, including a tractography, to better elucidate the hypothesis of anatomical variations. In our patient, despite the lack of definite MRI (DTI) and/or neurophysiological (MEP recording) evidence, the absence of clinical or radiological signs associated with pyramid decussation abnormalities suggested a normal corticospinal crossover (Figure 5).

It is known that hemiparesis due to compression of the contralateral peduncle is usually well proportioned on one side of the body. In contrast, hemiparesis due to motor cortex origin is disproportionate (predominates on the lower or upper limb) given the compression on different places of the motor homunculus [47,48]. Judging by the grade and topography of the IH in the presented case, a peduncular origin would be more likely. The hemiparesis is well proportioned on the right side of the body, with a right positive Babinski sign and spasticity. Thus, the probability of anatomical decussation disorder in the CST appears limited.

The possibility of diaschisis as a cause of IH in our case was considered. However, the theory of diaschisis implies the acute onset of symptoms, and although most of the time it was described in cerebrovascular lesions, cases of diaschisis following tumoral masses were often described [26]. Given that the onset of these symptoms was approximately 4 months before hospital admission and worsened over time, the hypothesis of diaschisis was unlikely. According to the concept of diaschisis, the neurological signs are discreet or absent in slow-growing tumors. In contrast, the presented patient’s symptoms were prominent, chronic, and worsened over time. Moreover, after the neurosurgical removal of the meningioma, the patient had full symptom remission. In patients with diaschisis, recovery might take time until the process wears off, and the regression of symptoms is correlated with neuroanatomical pathways in phases, starting from the lesional site in the brain.

**Figure 6:**
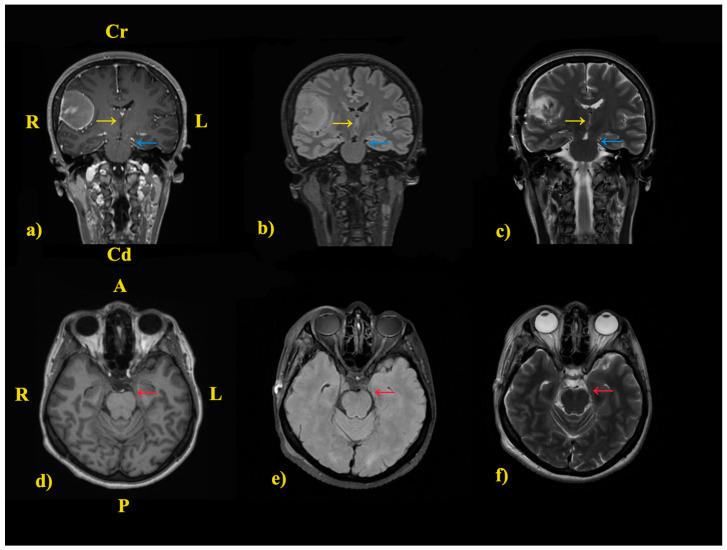
Suggestive preoperative MRI sequences: (**a**) Contrast-enhancement on T1-Weighted sequence, coronal projection. (**b**) T2 FLAIR sequence, coronal projection. (**c**) T2-Weighted sequence, coronal projection. (**d**) T1-Weighted sequence, axial projection. (**e**) T2 FLAIR sequence, axial projection. (**f**) T2-Weighted sequence, axial projection. Cr—Cranial; Cd—Caudal; R—Right; L—Left. The coronal projections show a left midline shift (yellow arrow), contact with the free tentorial edge, and compression on the left ambient cistern (blue arrow). The contact with the free tentorial edge and compression on the left ambient cistern are also visible on axial projections (red arrow).

The Kernohan-Woltman notch phenomenon and its particular type, the third frontal convolution syndrome, were also considered. The medical literature shows that approximately 98% of cases with IH are caused by KWNP, proven by neuroimaging, pathological, or even neurophysiological evidence studies [46]. The neuroimaging results showed that the right pterional meningioma created a mass effect in the presented patient due to its large volume, resulting in compression on the neighboring anatomical structures (Figure 6).

As a consequence of this compression, a midline shift was observed. Apart from the progressive midline shift, the cerebral peduncle’s subsequent compression on the tentorium’s free edge was present, and was most likely the cause of the IH. In addition, the possibility of a transitory compression is worth mentioning.

According to Adler and Milhorat, morphometric measurements of the anatomy of the tentorial notch can provide valuable information [49]. Therefore, we considered performing these measurements on our patient and analyzing the results afterward. The notch length (NL) was 63.39 mm, while the maximum notch width (MNW) was 33.15 mm. According to the authors’ classification scheme, our patient has a wide MNW, ranging between 32.0 and 39.0 mm, and a long NL, ranging between 62.0 and 70.0 mm. Thus, the type of notch in this case is large, predisposing her to neurological phenomena like KWNP.

It is important to mention that the tumoral mass is not associated with peripheral vasogenic edema. When present, vasogenic edema may contribute to both brain distortion (mass effect) and dysfunction of the surrounding brain. In lesions like meningiomas, edema is due to disturbed venous drainage of the neighboring healthy nervous tissue [50]. Although it has been stated [50] that peritumoral edema is correlated with more voluminous lesions, higher grades, higher invasiveness, and a higher recurrence rate, this was not the case in this patient.

Nevertheless, although the hypothesis of a disorder of anatomical decussation can not be excluded entirely since neuroimaging studies do not always reveal peduncular lesions, the smaller left ambient cistern suggests a compression in the area, specifically on the crus cerebri. The latest could be considered a significant reason supporting the hypothesis of KWNP. However, one of the most significant limitations of the current study was the absence of a tractography or DTI, given the initial unavailability in the hospital and the subsequent patient’s refusal. These advanced techniques could have provided more substantial evidence for ruling out CST anomalies and diaschisis. Given their potential, future studies, including them, would enhance knowledge and provide more insights regarding the IH.

Since the patient’s full recovery was observed after four weeks, the influence of neural plasticity should be mentioned since it has such a major impact. The phenomenon of neural plasticity refers to the ability of the nervous system to modify itself in response to an experience or an injury. The modifications are not only structural but also functional [51]. The mechanisms of neuroplasticity are represented by neuronal regeneration (collateral sprouting) and functional reorganization. Furthermore, it has been considered that this phenomenon has three phases. The first phase is seen in the first 2 days, depending on the mechanism of injury. In this stage, the initial events are represented by cellular death, which will impact various cortical pathways. Following the cell losses, the brain starts to use secondary networks to maintain its functions [52]. The second phase of neuroplasticity is observed in the following weeks when the brain recruits supporting cells and the cortical pathways shift from inhibitory to excitatory. During this stage, the formation of new neural connections and synaptic plasticity is observed [53]. Finally, the third phase can be seen in the following months. In this stage, a reorganization and axonal sprouting happen around the damaged area, and the brain continues to remodel itself [52]. Many factors, such as physical activity, the environment, repetition of tasks, motivation, neuromodulators, and medications, can influence neuroplasticity [53].

Although neuroplasticity can also have a negative impact in rare cases, in the presented case, it was beneficial. This process helped the patient restore motor function, which explains why the symptoms disappeared after 4 weeks. The short recovery time could be explained by the young age, lack of other medical conditions, and total removal of the injury factor.

The presented case was not necessarily a case report but provided a foundation for generating a stimulating and insightful discussion. The increase in reports in the last years, including original series of patients, supports the hypothesis that ipsilateral hemiparesis is not a forgotten or exceptional matter, but an underrated clinical phenomenon. In most cases, it can result in an incorrect diagnosis, given that it is a sign of a false localization. Although based on clinical examination, neuroimaging assessments, and the discussed etiological perspectives, the conclusion leans toward a peduncular cause of IH and the discussion remains open.

Finally, although modern neuroimaging studies can provide important insights into the causes of IH, none of these hypotheses proposed throughout the decades can satisfactorily account for the etiology. Nevertheless, this is the main remaining knowledge gap in understanding this paradoxical neurological finding [1]. Future research directions to further investigate its pathophysiology and optimal management strategies include a thorough neurological examination and exhaustive advanced neuroimaging and neurophysiological investigations.

## 4. Conclusions

The hypotheses behind IH were developed mainly from a historical theoretic point of view to explain this “paradoxical” phenomenon. The most discussed theories include the disorders of anatomical decussation of the CST, the concept of diaschisis, the Kernohan-Woltman notch phenomenon, and Ectors’ Syndrome. Although predisposing factors were observed, there is insufficient evidence to support or refute most correlations. To conclude the mechanism behind IH, thorough clinical examination and further research in neuroimaging assessment and functional studies are essential. When available, advanced neuroimaging techniques, such as tractography or functional imaging, could provide important information. Thus, more similar studies are encouraged.

## Figures and Tables

**Table 1:** Summary of neurological findings.

Neurological Examination	Findings
Cranial nerves	Normal
Sensation	Normal
Speech	Normal
Cognition	Normal
Comprehension and repetition	Normal
Motor examination	MRCS grade 4 for the right upper and lower limbs
Deep tendon reflexes	3+ (brisk response) on the right side
Pathologic reflexes	Right positive Babinski sign
Other	Headaches; generalized tonic-clonic seizures

**Table 2:** Inflammatory markers and coagulation studies in the presented patient.

Inflammatory Marker	Value (Normal Range)	Coagulation Tests/Markers	Value (Normal Range)
ESR	6 (3–11 mm/h)	APTT	29.50 (24.3–35 s)
WBC	6.3 (4.8—10.8 × 10^3^/uL)	Fibrinogen	240.27 (220–496 mg/dL)
CRP	0.309 (<5 mg/L)	PT	13.100 (11.8–15.1)
		INR	1.0178 (0.85–1.15)
		APP	78.596 (70–140%)

ESR—erythrocyte sedimentation rate; WBCs—white blood cells; CRP—C-Reactive Protein; APTT—activated partial thromboplastin time; PT—prothrombin time; INR—international normalized ratio; APP—prothrombin activity percentage.

**Table 3:** Summarization of the different pathophysiological mechanisms of IH.

Disorders of Anatomical Decussation	Diaschisis	Kernohan-Woltman Notch Phenomenon	The Syndrome of the Third Frontal Convolution
Lesions above the pyramidal decussation produce contralateral weakness, while those below the pyramidal decussation will produce ipsilateral weakness	Recovery is a passive process and can be more efficient in young patients than in older ones	Sudden and massive uncal herniation caused by a supratentorial mass lesion	The presence of intracranial hypertension, seizures +/− contralateral hemiparesis
The hemiparesis is disproportionate (predominates on the lower or upper limb)	Regresses in phases correlated to neuroanatomical pathways, starting from the lesional site in the brain	Hemiparesis due to compression of the contralateral peduncle is usually well proportioned on one side of the body	Frontal syndrome: Obtundation; instability; mental symptoms; language impairment
Advanced neuroimaging studies, such as DTI and tractography, can provide a certain diagnosis	Reduced activity in regions with diaschisis on advanced neuroimaging studies	Extensive midline shift due to mass effect, resulting in the indentation of the contralateral cerebral crus by the tentorium cerebelli	Radiological syndrome: angiographic engorgement of an anterior collateral artery from the anterior division of the middle meningeal artery, contralateral displacement of the ventricles on ventriculography
Partial or complete uncrossed pyramidal tracts on either side at the level of the medulla oblongata could be observed	The signs are prominent in acute injuries and are discreet/absent in chronic and slow-growing lesions	The grade of mass effect, the degree of brainstem displacement, anatomical variations of the tentorial incisura, and the width of the tentorial notch are considered contributing factors	Lateral displacement of the diencephalic-brainstem axis and compression of the contralateral cerebral peduncle against the free tentorial border
Independent laterality/motor dominance	Dependent laterality/motor dominance	Independent laterality/motor dominance	Independent laterality/motor dominance
The mass effect is unnecessary	The mass effect is unnecessary	The mass effect is necessary	The mass effect is necessary
Independent tentorial anthropometry	Independent tentorial anthropometry	Tentorial anthropometry may play a role	Tentorial anthropometry may play a role
Motor deficit distribution is dependent on primary lesion topography	Motor deficit distribution is dependent on primary lesion topography	Motor deficit distribution is mostly crural	Brodmann area 8 motor syndrome: Conjugated head rotation and eye deviation towards the lesion, motor discoordination, and pseudo parkinsonism
Other NS anomalies frequent	Independent of other NS anomalies	Independent of other NS anomalies	Independent of other NS anomalies

NS—nervous system.

## Data Availability

Additional patient data can be obtained from the author upon reasonable request. The data are not publicly available due to privacy concerns.
